# Post-operative Right Ventricular Failure After Cardiac Surgery: A Cohort Study

**DOI:** 10.3389/fcvm.2021.667328

**Published:** 2021-06-14

**Authors:** David Levy, Driss Laghlam, Philippe Estagnasie, Alain Brusset, Pierre Squara, Lee S. Nguyen

**Affiliations:** Intensive Care Medicine Department, CMC Ambroise Paré, Neuilly-sur-Seine, France

**Keywords:** right ventricular failure, cardiac surgery, nitric oxide, sildenafil, cohort analyses

## Abstract

**Introduction:** Right ventricular failure (RVF) after cardiac surgery is an important risk factor for morbidity and mortality. Its diagnosis is challenging, and thus, its incidence and predictors are not well-established. We investigated the incidence, complications, and variables associated with clinically relevant post-operative RVF.

**Methods:** We included all patients who underwent cardiac surgery with cardiopulmonary bypass between 2016 and 2019 in a cardiac surgery center with standardized diagnostic and therapeutic management of RVF. RVF was considered only if clinically relevant: associated with hemodynamic instability requiring catecholamine support and inhaled nitric oxide relayed by sildenafil.

**Results:** Overall, 3,826 patients were included, of whom, 110 (2.9%) developed post-operative RVF. Mortality was not different among patients who developed post-operative RVF, compared with the rest of the cohort (1.8 vs. 0.7%, *p* = 0.17). Using a composite outcome that combined death, reintubation, stroke, and prolonged intensive care unit stay (more than 14 days) yielded an incidence of 6.6%, and RVF was associated with this composite outcome with an odds ratio of 3.6 (2.2–5.8), *p* < 0.001. In a multivariable model, pre-operative variables independently associated with post-operative RVF were pre-operative atrial fibrillation (AF) {adjusted odds ratio (adjOR) 3.22 [95% confidence interval (95%CI) = 1.94–5.36], *p* < 0.001}, left ventricle ejection fraction below 50% [adjOR = 2.55 (95%CI = 1.52–4.33), *p* < 0.001], systolic pulmonary artery pressure above 55 mmHg [adjOR = 8.64 (95%CI = 5.27–14.1); *p* < 0.001], mitral valve surgery [adjOR = 2.17 CI (95%CI = 1.28–3.66), *p* = 0.004], and tricuspid valve surgery [adjOR = 10.33 (95%CI = 6.14–17.4), *p* < 0.001]. In patients who developed post-operative RVF requiring treatment, 32 (29.1%) showed RV dysfunction before surgery.

**Conclusion:** In this cohort study, 2.9% of patients developed clinically significant post-operative RVF. Moreover, RVF was associated with severe adverse outcomes, including death, strokes, reintubation, and prolonged intensive care unit stay.

## Introduction

Right ventricular failure (RVF) after cardiac surgery has been found as a significant cause of morbidity and mortality in several settings (1). Mechanisms of RVF include suboptimal myocardial protection during surgery, long cardiopulmonary bypass (CPB) time, right ventricular (RV) myocardial ischemia or infarction, atrial arrhythmias or loss of atrioventricular synchrony, reperfusion lung injury with secondary pulmonary hypertension (PH), post-operative pulmonary micro- or macro-embolism, pre-existing pulmonary vascular disease, protamine-induced PH, and others (2). An acute increase in PH leads to higher RV afterload, altered RV-pulmonary artery coupling, and then RV failure with compromised hemodynamic (3). Pulmonary vascular resistance (PVR) may increase after CPB because of atelectasis, ischemia–reperfusion injury with inflammatory cytokines, endothelial damage, and reduction of nitric oxide release (4, 5).

Post-operative RVF management includes pulmonary vasodilators, optimal fluid management, inotrope support, and, when necessary, restoring a compromised RV myocardial perfusion pressure, systemic vasopressors. As of yet, these treatments are not supported by robust studies. Inhaled nitric oxide (iNO) has been approved for persistent PH of the newborn but is used off-label in cardiac post-operative RV failure despite poor evidence in hard clinical outcomes (6–8). Similarly, sildenafil showed promise in clinical studies, in post-operative care, by reducing pulmonary pressures and resistance with no alteration in systemic hemodynamic and oxygenation (9, 10).

Another issue lies in the lack of a consensual definition of RVF after surgery (11). Consequently, the incidence of RVF and its risk factors are not well-established. Although some reports described risk scores of RVF after left ventricular assist device implantation, the literature is poor in standard cardiac surgery (12, 13).

This study aimed to characterize the incidence, outcomes, and predictors of RVF after cardiac surgery.

## Patients and Methods

### Study Population and Design

From January 1, 2016, to December 31, 2019, the study included all adult patients undergoing cardiac operations with CPB. The exclusion criteria were age below 18 years. We performed a retrospective analysis of data collected prospectively, including perioperative characteristics, post-operative drugs, and outcomes. Data were anonymized as per national regulation and used with the approval of an institutional review board committee (Commission Nationale de l'Informatique et des Libertés, CNIL, declaration number 2109982, 17/10/2017). All the data are part of the Registry for the Improvement of Post-operative Outcomes in Cardiac and Thoracic Surgery database (NCT03209674). Patients' opposition to the use of anonymized data by investigators was systematically sought (i.e., informed consent was obtained from all patients).

### Definitions

Post-operative RVF was defined by the combination of (1) hemodynamic instability requiring administration of vasopressors and/or inotropes and (2) immediate post-operative needs of pulmonary vasodilators (iNO followed by sildenafil® per os). In the post-operative period, patients were systematically explored with echocardiography when presenting with hemodynamic instability. RVF required hypokinesia of the RV free wall or RV/left ventricle (LV) interdependence or enlarged RV with RV/LV ratio > 1. Additional optional parameters included tricuspid annular plane systolic excursion (TAPSE) <16 mm and tissue doppler imaging with peak myocardial velocity at the lateral tricuspid annulus (S wave) <9.5 cm/s, also considered if patients did not undergo tricuspid intervention.

### Right Ventricular Failure Management

Inhaled NO was initiated at RVF diagnosis. It was administered at 10 ppm and once hemodynamic stability was achieved for at least 24 h; weaning was achieved by decreasing 1 ppm every hour. Sildenafil was initiated during the first 48 h after surgery, whenever oral intake was possible, with a dosage of 20 mg every 8 h.

### Statistical Analysis

Data distribution was assessed using the Kolmogorov–Smirnov-test with Lilliefors correction. Continus variables were summarized as mean ± standard deviation or median (interquartile range), as appropriate, and were compared, using the Student *t*-test or Mann–Whitney *U*-test accordingly. Categorical variables were presented as count (percentage) and compared using the chi-square or Fisher exact-test, as appropriate.

Binary logistic regression analysis was performed to assess variables independently associated with RVF. The multivariable model was built by the stepwise selection, with an entry criterion of *p* < 0.10. Variables included in the model were age, sex, previous AF, estimated glomerular filtration rate, systolic pulmonary arterial pressure (sPAP) > 55 mmHg, left ventricular ejection fraction (LVEF) <50%, mitral surgery, tricuspid intervention, and CPB length.

Comparing patients who presented post-operative RVF and those who did not, regarding mortality, was performed using proportion comparison, and using survival analysis, censored at 14 days, with Kaplan–Meier estimates. This censorship was used because 97% of patients were discharged from the intensive care unit (ICU) by day 14, and the remaining 3% presented heterogeneous length of stay, precluding from linear assumptions. For sensitivity, uncensored analyses were performed.

All statistical analyses were performed using IBM SPSS version 26.0 (IBM Corporation, Armonk, New York). A two-sided *p*-value <0.05 was considered to be statistically significant. Figures were drawn using Prism 8.4.3 (GraphPad Software, California, USA) and R software 3.6 (R project, worldwide community software).

## Results

### Patients and Baseline Characteristics

We included 3,826 consecutive patients who underwent cardiac surgery with CPB. Baseline characteristics are shown in Table 1.

**Table 1:** Baseline characteristics.

	**Total cohort (*n* = 3,716)**	**RVF group (*n* = 110)**	***p*-value**
Age (years)	68.6 (±10.9)	71.0 (±9.9)	0.018
Female	935 (25.2%)	40 (36.4%)	0.008
BMI (kg/m^2^)	26.6 (±4.6)	25.1 (±4.9)	0.001
COPD	282 (7.6%)	13 (11.8%)	0.1
Hypertension	2,008 (54.2%)	41 (37.3%)	<0.001
Type II diabetes	996 (26.9%)	22 (20.0%)	0.1
Previous AF	287 (8.4%)	28 (29.2%)	<0.001
eGFR (ml/min)	69.6 (57.0–82.7)	57.7 (44.5–71.9)	<0.001
Dialysis (>3 months)	48 (1.3%)	5 (4.5%)	0.017
Previous cardiac surgery	342 (9.2%)	23 (20.9%)	<0.001
Euroscore II	1.7 (0.97–3.2)	4.1 (2.0–8.0)	<0.001
**Pre-operative LVEF**			
*30–49%*	470 (12.7%)	25 (23.1%)	0.001
* <30%*	68 (1.8%)	3 (2.8%)	0.45
sPAP 35–55 mmHg	850 (22.9%)	30 (33.7%)	0.017
sPAP > 55 mmHg	164 (4.4%)	49 (50.5%)	<0.001
**Type of surgery**			
CABG	2,221 (59.9%)	22 (20.0%)	<0.001
Mitral valve	621 (16.7%)	71 (64.5%)	<0.001
Aortic valve	1,190 (32.1%)	18 (16.4%)	<0.001
Tricuspid valve	135 (3.6%)	55 (50.0%)	<0.001
CPB duration (minutes)	79.3 (±29.8)	96.4 (±34.0)	<0.001
Aortic clampage time (minutes)	61.7 (±23.0)	63.4 (±24.7)	0.16

*Categorical variables are presented as number (proportion) and continuous variables as mean (standard deviation)*.

*AF, atrial fibrillation; BMI, body mass index; CABG, coronary artery bypass graft; CPB, cardiopulmonary bypass; COPD, chronic obstructive pulmonary disease; eGFR, estimated glomerular filtration rate; sPAP, systolic pulmonary arterial pressure*.

Post-operative RVF occurred in 110 patients (2.9%). Compared with the group who did not develop RVF, patients in the RVF group were older (71.0 ± 9.9 vs. 68.6 ± 10.9 years, *p* = 0.018), had more women (36.4 vs. 25.2%, *p* = 0.008), more history of AF (29.2 vs. 8.4%, *p* < 0.001), lower pre-operative estimated glomerular filtration rate [57.7 (44.5–71.9) vs. 69.6 (57.0– 82.7) ml/min per 1.73 m^2^, *p* < 0.001], and higher EuroScore II [4.1 (2.0–8.0) vs. 1.7 (0.97–3.2), *p* < 0.001]. Regarding pre-operative echocardiographic data, patient in the RVF group presented more pre-operative LVEF below 50% (25.5 vs. 14.5%, *p* = 0.001) and elevated sPAP above 55 mmHg (50.5 vs. 4.4%, <0.001).

The type of surgical procedure affected the risk of developing RVF. In particular, mitral and tricuspid valve procedures were more at risk (64.5 vs.16.7%, *p* < 0.001, and 50 vs. 3.6%, *p* < 0.001, respectively). CPB time was also higher in patients who developed post-operative RVF (96.4 ± 34.0 vs. 79.3 ± 29.8 min, *p* < 0.001, respectively).

### Clinical Outcomes and Prognosis After Right Ventricular Failure

**Figure 1:**
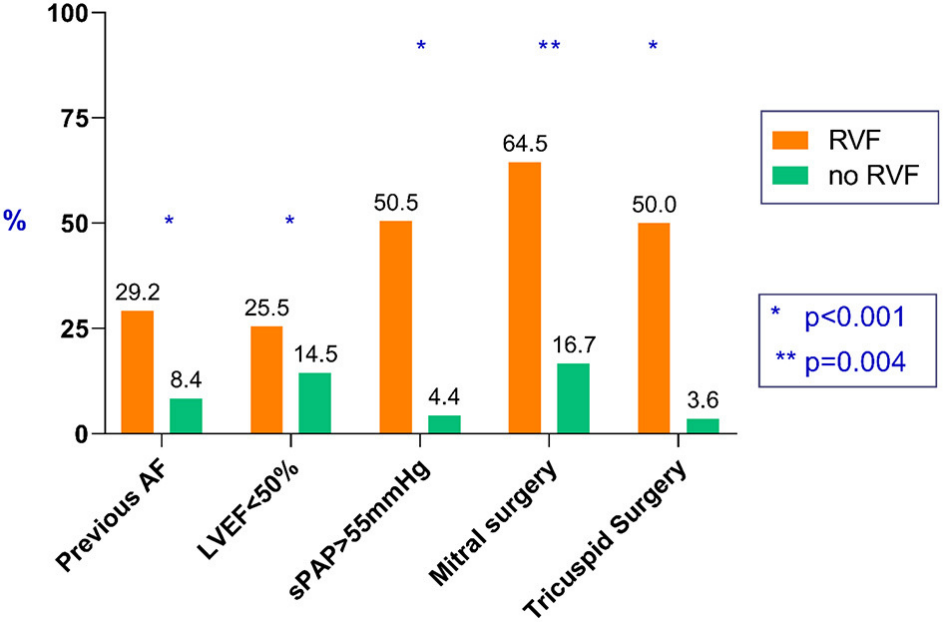
Variables associated with post-operative right ventricular failure (RVF).

Post-operative outcomes were similar in patients who presented RVF and those who did not, regarding mortality in ICU (RVF and those without RVF, respectively, 1.8 vs. 0.7%, *p* = 0.17), mechanical ventilation duration [4 (3–6) vs. 4 (3–6) h, *p* = 0.14], veno-arterial extracorporeal membranous oxygenation requirement (0.9 vs. 0.3%, *p* = 0.29), and stroke (0 vs. 1.7%, *p* = 0.26) (see Figure 1 and Table 2).

**Table 2:** Post-operative outcomes.

	**Total cohort (*n* = 3,716)**	**RVF group (*n* = 110)**	***P***
Death in ICU	26 (0.7%)	2 (1.8%)	0.17
Mechanical ventilation time (hours)	4 (3–6)	4 (3–6)	0.14
VA-ECMO	11 (0.3%)	1 (0.9%)	0.29
Stroke	63 (1.7%)	0	0.26
Post-operative AF	973 (26.3%)	65 (59.1%)	<0.001
ICU length of stay (days)	3 (3–4)	4(3–9)	<0.001

*Categorical variables are presented as number (proportion) and continuous variables as mean (standard deviation)*.

*AF, atrial fibrillation; ICU, intensive care unit; RVF, right ventricular failure; VA-ECMO, veno-arterial extracorporeal membranous oxygenation*.

The outcomes that were different were ICU length of stay (longer in patients with RVF), with 4 (3–9) vs. 3 (3–4) days (*p* < 0.001), and incidence of AF with 59.1 vs. 26.3% (*p* < 0.001).

**Figure 2:**
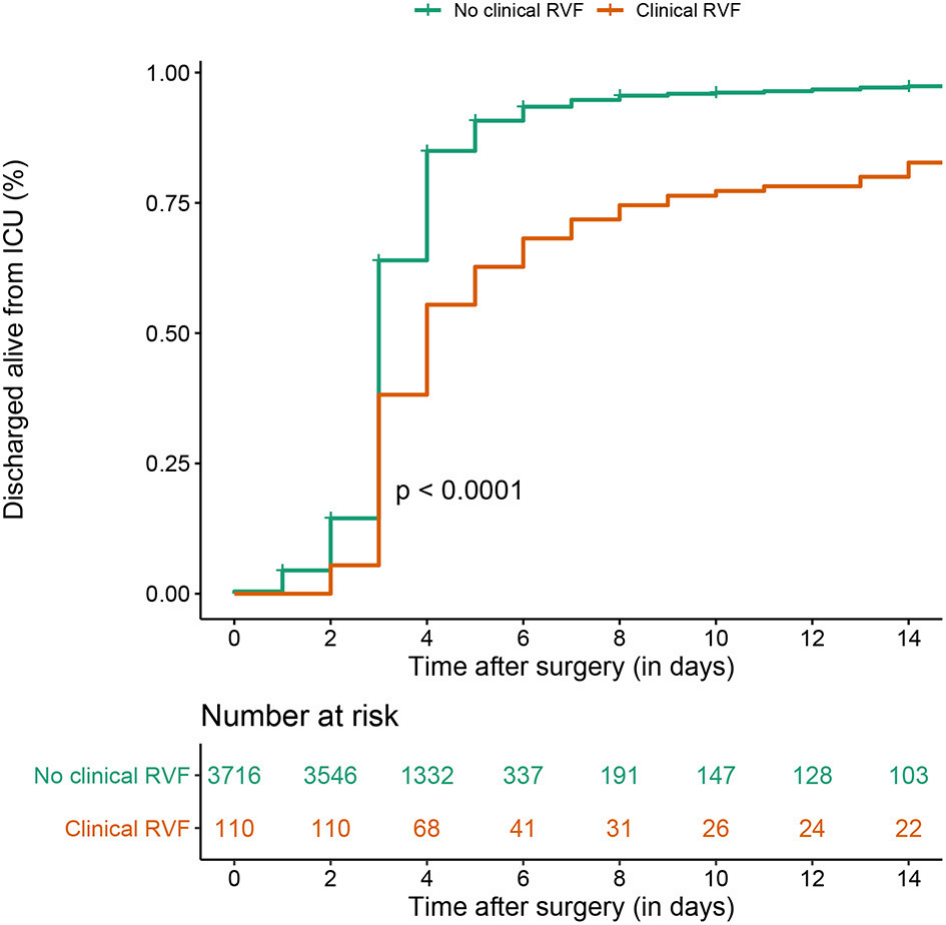
Kaplan–Meier estimate of ICU discharge alive, censored at 14 days between clinical post-operative RVF and control patients in the overall surgery cohort.

Focusing on the 14-day in-hospital follow-up, patients who developed post-operative RVF were less likely to be discharged at this point than patients who did not present RVF (log-rank *p* < 0.0001, see Figure 2). The uncensored analysis yielded a similar outcome between patients with or without post-operative RVF (log-rank *p* = 0.70).

For sensitivity, we computed a composite outcome that combined death, reintubation, stroke, and prolonged ICU stay (more than 14 days). We found an incidence of 252/3,826 (6.6%), and RVF was associated with this composite outcome (19.1 vs. 6.2%, odds ratio = 3.6 (2.2–5.8), *p* < 0.001).

### Variables Associated With the Incidence of Right Ventricular Failure

In a logistic regression multivariable model, variables independently associated post-operative RVF were pre-operative AF {adjusted odds-ratio (adjOR) 3.22 [95% confidence interval (95%CI) = 1.94–5.36], *p* < 0.001}, left ventricle ejection fraction below 50% [adjOR = 2.55 (95%CI = 1.52–4.33), *p* < 0.001], systolic pulmonary artery pressure above 55 mmHg [adjOR = 8.64 (95%CI = 5.27–14.1); *p* < 0.001], mitral valve surgery [adjOR = 2.17 CI (95%CI = 1.28–3.66), *p* = 0.004], and tricuspid valve surgery [adjOR = 10.33 (95%CI = 6.14–17.4), *p* < 0.001] (see Table 3).

**Table 3:** Variables associated with post-operative right ventricular failure.

	**Univariate Model**	**Multivariate Model**
	**OR (95%CI), *p*-value**	**OR (95%CI), *p*-value**
Age	1.02 (1.01–1.04), 0.02	
Female	1.71 (1.16–2.55), 0.007	
COPD	1.63 (0.90–2.95), 0.11	
Previous AF	4.5 (2.88–7.17), <0.001	3.22 (1.94–5.36), <0.001
eGFR	0.98 (0.97–0.99), <0.001	
LVEF <30%	1.52 (0.47–4.9), 0.48	
LVEF <50%	2.02 (1.30–3.13), 0.002	2.55 (1.52–4.33), 0.001
sPAP > 55 mmHg	23.11 (15.04–35.49), <0.001	8.64 (5.27–14.1), <0.001
Mitral surgery	9.19 (6.16–13.71), <0.001	2.17 (1.28–3.66), 0.004
Tricuspid surgery	28.04 (18.55–42.4), <0.001	10.33 (6.14–17.4), <0.001
CPB duration	1.01 (1.01–1.02), <0.001	
Aortic clampage	1.00 (0.99–1.01), 0.48	

*Variables included in the multivariable model: age, sex, previous AF, eGFR, sPAP > 55 mmHg, LVEF <50%, mitral surgery, tricuspid intervention, and CPB length*.

*AF, atrial fibrillation; CPB, cardiopulmonary bypass; COPD, chronic obstructive pulmonary disease; eGFR, estimated glomerular filtration rate; LVEF, left ventricular ejection fraction; sPAP, systolic pulmonary artery pressure*.

### Pre-operative Right Ventricular Function in Patients With Post-operative Right Ventricular Failure

Focusing in patients who presented RVF (*n* = 110), pre-existing RV dysfunction [with either TAPSE <16 mm and tissue doppler imaging with peak myocardial velocity at the lateral tricuspid annulus (S wave) <9.5 cm/s] was found in 32 (29.1%) patients (Table 4). Compared with those with preserved pre-operative RV function, those with pre-operative RV dysfunction showed more moderate to severe TR (81.3 vs. 38.2%, *p* < 0.001), larger RA surface [26.5 (22.3–31.8) vs. 21 (14–23) cm^2^, *p* < 0.001] and underwent more frequently tricuspid procedures (65.6 vs. 43.4%, *p* = 0.035). Inhaled NO tended to used more frequently in patients with pre-operative RVF (87.5 vs. 69.7%, *p* = 0.05).

**Table 4:** Comparison between patients with normal pre-operative RV function and patients with pre-operative RV dysfunction, in the subgroup of patients presenting post-operative RV dysfunction (*n* = 110).

	**Normal pre-operative RV function (*n* = 78)^†^**	**Pre-operative RV dysfunction (*n* = 32)**	***P***
TAPSE (mm)	22 (21–24)	15 (14–15.25)	–
S wave (cm/s)	13 (13–14)	9 (8–10)	–
TR, moderate to severe	29 (38.2%)	26 (81.3%)	<0.001
Right atrium surface (cm^2^)	21 (14–23)	26.5 (22.3–31.8)	<0.001
Previous AF	21 (27.6%)	14 (43.8%)	0.1
LVEF <50%	19 (25%)	8 (25%)	1
sPAP > 55 mmHg	30 (39.5%)	17 (53.1%)	0.19
Mitral surgery	49 (64.5%)	20 (62.5%)	0.85
Tricuspid surgery	33 (43.4%)	21 (65.6%)	0.035

*^‡^Pre-operative echocardiographic data were incomplete for two patients*.

*Categorical variables are presented as number (proportion) and continuous variables as mean (standard deviation)*.

*AF, atrial fibrillation; LVEF, left ventricular ejection fraction; sPAP, systolic pulmonary artery pressure; TR, tricuspid regurgitation; TAPSE, tricuspid annular plane systolic excursion*.

## Discussion

In this study, we described the incidence, risk factors, and prognosis of post-operative RVF. We observed that (1) clinically significant RVF after cardiac surgery is rare, occurring below 3%; (2) despite higher predicted perioperative risk (i.e., EuroScore II), treatment with iNO and sildenafil allowed to reach similar post-operative results regarding hard clinical outcomes when comparing patients with post-operative with and without RVF; (3) independent variables associated with post-operative RVF included the history of AF, pre-operative LVEF below 50%, mitral surgery, tricuspid surgery, and pre-operative sPAP above 55 mmHg; (4) in patients who presented post-operative RVF, its severity was associated with pre-operative RV dysfunction.

Post-operative RVF in cardiac surgery ICU is a major concern because of its challenging diagnosis: sternotomy is an obstacle to procure accurate and reproducible information regarding echocardiographic right heart evaluation. Due to the standard approach in cardiac surgery with pericardial opening, TAPSE may be inherently reduced in the post-operative period without patent clinical RVF (14). Moreover, TAPSE after tricuspid annuloplasty may lead to an underestimation of RV systolic function (15). Definition of RVF is heterogeneous among physicians in the field and the literature, going from only echocardiographic definitions to mild or very severe circulatory failure (16, 17). Although echocardiography is an overall powerful tool, in the post-operative setting, it presents limitations, the first of which is a possible lack of sonographic window allowing for accurate measurements, particularly in mechanically ventilated patients (18). Meanwhile, a single echocardiographic parameter may not be sufficient to define RVF, and as echocardiographic assessment of longitudinal RV systolic function such as TAPSE and S wave may be eyeballed by expert visual assessment (19), we chose a pragmatic endpoint defined as echocardiographic assessment RVF combined with clinical hemodynamic instability requiring immediate treatment.

Our data could not allow for the analysis of the effect of iNO and sildenafil, as RVF treatment, because of a lack of a control group. Recent studies showed interest in sildenafil administration on its own or in association with iNO. In our experience, combining both drugs in the post-operative period seemed to be safe and efficient in decreasing both pressure and resistance in the pulmonary circulation without affecting cardiac output, although, as of yet, no study showed differences in clinical outcomes (20–22).

We confirmed that mitral surgery and previous LVEF <50% were associated with post-operative RVF, as already described previously, with a high incidence of low cardiac output state after mitral valve procedures (23, 24). The principal mechanism invoked is an abrupt increase in afterload due to elimination of the reverse flow into the left atrium, all the more so if LV was decreased before correction (25, 26).

As we observed, previous AF could be associated with a complicated post-operative course (27). Gorter et al. showed in patients with heart failure with preserved LVEF that RV and RA functions were more depressed in patients with AF and patients in sinus rhythm but with prior AF, compared with patients without any history of AF (28). Also, they suggested these findings were independent of pulmonary pressures. According to recent studies, functional TR is associated with right atrium (RA) enlargement in the setting of chronic AF (29). Thus, AF, tricuspid regurgitation, and RA enlargement are somehow working together, participating in the development of RVF. Then, TR correction could represent a key therapeutic option, but it has shown to be a major risk factor for post-operative RVF (30, 31). Before surgery, the RV adapted to chronic volume overload, but surgical TR correction could lead to an abrupt increase in afterload with reduced pre-load and then uncoupling RV and pulmonary circulation as an analogy with LV hemodynamics in mitral valve closure, but because of RV anatomy and physiology, it would not support such hemodynamic modification as the LV could accept it. Besides, CPB with induced RH and abnormal septal motion has shown deleterious effect in RV hemodynamics (2). Finally, severe pre-operative RH is a well-known risk factor for post-operative RVF, corresponding to our findings with a threshold of sPAP > 55 mmHg in our study. We acknowledge that this study does not solve the issue of whether iNO and sildenafil are appropriate to treat RVF, but it comforts the fact that RVF may be anticipated in some procedures (mainly tricuspid and mitral valve surgery) and that these treatments seem to alleviate the poor prognosis of an RVF outcome.

We acknowledge several limitations. First, although we chose criteria that satisfied clinician need for post-operative RVF (i.e., which required specific treatment, including vasopressors and inhaled NO), there are no consensual and easily obtainable RVF criteria in clinical practice. Secondly, lack of difference in mortality may be imputed to a lack of power; all the more so that the computation of a composite outcome which combined deaths, strokes, prolonged ICU stay, and reintubation showed significantly increased risk in patients who developed post-operative RVF. Third, our center only performed standard cardiac surgery excluding congenital cardiac procedures, heart transplantation, or LVAD implantations, all considered at high risk for post-operative RVF. Fourth, pre-operative RV assessment parameters were only retrievable in patients who developed post-operative RVF; thus, we could not integrate these elements into multivariable analysis regarding pre-operative risk factors of developing post-operative RVF. Nevertheless, we managed to study pre-operative RV function in patients with post-operative RVF, providing important insight into this subgroup in favor of a link between RV dysfunction, AF, TR, and enlarged RA.

Finally, we acknowledge that the lack of a control group (i.e., patients who presented post-operative RVF but were treated with neither iNO nor sildenafil) makes any analysis of treatment efficacy impossible.

Thus, we advocate for the design of a dedicated randomized controlled trial to address this rare but severe issue that is post-operative RVF.

## Conclusion

Post-operative RVF occurred in 2.9% of patients after cardiac surgery. Variables significantly associated with RVF were history of AF, LVEF <50%, sPAP > 55 mmHg, and mitral and tricuspid valve procedures.

## Data Availability Statement

The raw data supporting the conclusions of this article will be made available by the authors, without undue reservation.

## Ethics Statement

Ethical review and approval was not required for the study on human participants in accordance with the local legislation and institutional requirements. The ethics committee waived the requirement of written informed consent for participation.

## Author Contributions

DLe wrote the initial manuscript, participated to data collection, and performed statistical analyses. DLa, PE, AB, and PS participated to data collection and provided critical review. AB also curated the database. LN supervised the study, participated to data collection, analyses, and wrote the final manuscript. All authors contributed to the article and approved the submitted version.

## Conflict of Interest

The authors declare that the research was conducted in the absence of any commercial or financial relationships that could be construed as a potential conflict of interest.

## References

[1] Denault AY Pearl RG Michler RE Rao V Tsui SSL Seitelberger R. Tezosentan and right ventricular failure in patients with pulmonary hypertension undergoing cardiac surgery: the TACTICS trial. J Cardiothorac Vasc Anesth. (2013) 27:1212–7. 10.1053/j.jvca.2013.01.023 23523254

[2] Haddad F Couture P Tousignant C Denault AY. The right ventricle in cardiac surgery, a perioperative perspective: II. Pathophysiol Clin Import Manage Anesth Analg. (2009) 108:422–33. 10.1213/ane.0b013e31818d8b92 19151265

[3] Vonk Noordegraaf A Chin KM Haddad F Hassoun PM Hemnes AR Hopkins SR. Pathophysiology of the right ventricle and of the pulmonary circulation in pulmonary hypertension: an update. Eur Respir J. (2019) 53:1801900. 10.1183/13993003.01900-2018 30545976 PMC6351344

[4] Morita K Ihnken K Buckberg GD Sherman MP Ignarro LJ. Pulmonary vasoconstriction due to impaired nitric oxide production after cardiopulmonary bypass. Ann Thorac Surg. (1996) 61:1775–80. 10.1016/0003-4975(96)00146-4 8651783

[5] Gianetti J Del Sarto P Bevilacqua S Vassalle C De Filippis R Kacila M. Supplemental nitric oxide and its effect on myocardial injury and function in patients undergoing cardiac surgery with extracorporeal circulation. J Thorac Cardiovasc Surg. (2004) 127:44–50. 10.1016/j.jtcvs.2002.08.001 14752411

[6] Germann P Braschi A Della Rocca G Dinh-Xuan AT Falke K Frostell C. Inhaled nitric oxide therapy in adults: European expert recommendations. Intensive Care Med. (2005) 31:1029–41. 10.1007/s00134-005-2675-4 15973521

[7] Pepke-Zaba J Higenbottam TW Dinh-Xuan AT Stone D Wallwork J. Inhaled nitric oxide as a cause of selective pulmonary vasodilatation in pulmonary hypertension. Lancet Lond Engl. (1991) 338:1173–4. 10.1016/0140-6736(91)92033-X 1682593

[8] Fullerton DA Jones SD Jaggers J Piedalue F Grover FL McIntyre RC. Effective control of pulmonary vascular resistance with inhaled nitric oxide after cardiac operation. Cardiovasc Surg. (1996) 111:11. 10.1016/S0022-5223(96)70335-5 8614135

[9] Haché M Denault A Bélisle S Robitaille D Couture P Sheridan P. Inhaled epoprostenol (prostacyclin) and pulmonary hypertension before cardiac surgery. J Thorac Cardiovasc Surg. (2003) 125:642–9. 10.1067/mtc.2003.107 12658208

[10] Fattouch K Sbraga F Sampognaro R Bianco G Gucciardo M Lavalle C. Treatment of pulmonary hypertension in patients undergoing cardiac surgery with cardiopulmonary bypass: a randomized, prospective, double-blind study. J Cardiovasc Med Hagerstown Md. (2006) 7:119–23. 10.2459/01.JCM.0000203850.97890.fe 16645371

[11] Haddad F Couture P Tousignant C Denault AY. The right ventricle in cardiac surgery, a perioperative perspective: I. Anat Physiol Assess Anesth Analg. (2009) 108:407–21. 10.1213/ane.0b013e31818f8623 19151264

[12] Drakos SG Janicki L Horne BD Kfoury AG Reid BB Clayson S. Risk factors predictive of right ventricular failure after left ventricular assist device implantation. Am J Cardiol. (2010) 105:1030–5. 10.1016/j.amjcard.2009.11.026 20346326

[13] Bellavia D Iacovoni A Scardulla C Moja L Pilato M Kushwaha SS. Prediction of right ventricular failure after ventricular assist device implant: systematic review and meta-analysis of observational studies. Eur J Heart Fail. (2017) 19:926–46. 10.1002/ejhf.733 28371221

[14] Zanobini M Saccocci M Tamborini G Veglia F Di Minno A Poggio P. Postoperative echocardiographic reduction of right ventricular function: is pericardial opening modality the main culprit? BioMed Res Int. (2017) 2017:4808757. 10.1155/2017/4808757 28589141 PMC5446880

[15] de Agustin JA Martinez-Losas P de Diego JJG Mahia P Marcos-Alberca P Nuñez-Gil IJ. Tricuspid annular plane systolic excursion inaccuracy to assess right ventricular function in patients with previous tricuspid annulopasty. Int J Cardiol. (2016) 223:713–6. 10.1016/j.ijcard.2016.08.276 27573594

[16] Mehra MR Park MH Landzberg MJ Lala A Waxman AB International Right Heart Failure Foundation Scientific Working Group. Right heart failure: toward a common language. J Heart Lung Transplant. (2014) 33:123–6. 10.1016/j.healun.2013.10.015 24268184

[17] Vieillard-Baron A Naeije R Haddad F Bogaard HJ Bull TM Fletcher N. Diagnostic workup, etiologies and management of acute right ventricle failure: a state-of-the-art paper. Intensive Care Med. (2018) 44:774–90. 10.1007/s00134-018-5172-2 29744563

[18] Price S Prout J Jaggar SI Gibson DG Pepper JR. ‘Tamponade' following cardiac surgery: terminology and echocardiography may both mislead. Eur J Cardio-Thorac Surg. (2004) 26:1156–60. 10.1016/j.ejcts.2004.08.020 15541977

[19] Schneider M Aschauer S Mascherbauer J Ran H Binder C Lang I. Echocardiographic assessment of right ventricular function: current clinical practice. Int J Cardiovasc Imaging. (2019) 35:49–56. 10.1007/s10554-018-1428-8 30191507 PMC6373282

[20] Ram E Sternik L Klempfner R Eldar M Goldenberg I Peled Y. Sildenafil for pulmonary hypertension in the early postoperative period after mitral valve surgery. J Cardiothorac Vasc Anesth. (2019) 33:1648–56. 10.1053/j.jvca.2018.12.023 30685151

[21] Matamis D Pampori S Papathanasiou A Papakonstantinou P Tsagourias M Galiatsou E. Inhaled NO and sildenafil combination in cardiac surgery patients with out-of-proportion pulmonary hypertension: acute effects on postoperative gas exchange and hemodynamics. Circ Heart Fail. (2012) 5:47–53. 10.1161/CIRCHEARTFAILURE.111.963314 22057829

[22] Michelakis E Tymchak W Lien D Webster L Hashimoto K Archer S. Oral sildenafil is an effective and specific pulmonary vasodilator in patients with pulmonary arterial hypertension: comparison with inhaled nitric oxide. Circulation. (2002) 105:2398–403. 10.1161/01.CIR.0000016641.12984.DC 12021227

[23] Appelbaum A Kouchoukos NT Blackstone EH Kirklin JW. Early risks of open heart surgery for mitral valve disease. Am J Cardiol. (1976) 37:201–9. 10.1016/0002-9149(76)90313-1 1246953

[24] Sethi GK Miller DC Souchek J Oprian C Henderson WG Hassan Z. Clinical, hemodynamic, and angiographic predictors of operative mortality in patients undergoing single valve replacement. J Thorac Cardiovasc Surg. (1987) 93:884–97. 10.1016/S0022-5223(19)37049-7 3573798

[25] Ahmadi A Spillner G Johannesson T. Hemodynamic changes following experimental production and correction of acute mitral regurgitation with an adjustable ring prosthesis. Thorac Cardiovasc Surg. (1988) 36:313–9. 10.1055/s-2007-1022972 3232132

[26] Rankin JS Nicholas LM Kouchoukos NT. Experimental mitral regurgitation: effects on left ventricular function before and after elimination of chronic regurgitation in the dog. J Thorac Cardiovasc Surg. (1975) 70:478–88. 10.1016/S0022-5223(19)40322-X 1165639

[27] Greenberg JW Lancaster TS Schuessler RB Melby SJ. Postoperative atrial fibrillation following cardiac surgery: a persistent complication. Eur J Cardiothorac Surg. (2017) 52:665–72. 10.1093/ejcts/ezx039 28369234

[28] Gorter TM van Melle JP Rienstra M Borlaug BA Hummel YM van Gelder IC. Right heart dysfunction in heart failure with preserved ejection fraction: the impact of atrial fibrillation. J Card Fail. (2018) 24:177–85. 10.1016/j.cardfail.2017.11.005 29197548

[29] Cho JY Kim KH Kim JY Sim DS Yoon HJ Yoon NS. Predictors of reversible severe functional tricuspid regurgitation in patients with atrial fibrillation. J Cardiol. (2016) 68:419–25. 10.1016/j.jjcc.2015.11.010 26993263

[30] Spratt JA Olsen CO Tyson GS Glower DD Davis JW Rankin JS. Experimental mitral regurgitation. Physiological effects of correction on left ventricular dynamics. J Thorac Cardiovasc Surg. (1983) 86:479–89. 10.1016/S0022-5223(19)39112-3 6621079

[31] Suri RM Schaff HV Dearani JA Sundt TM Daly RC Mullany CJ. Determinants of early decline in ejection fraction after surgical correction of mitral regurgitation. J Thorac Cardiovasc Surg. (2008) 136:442–7. 10.1016/j.jtcvs.2007.10.067 18692655

